# Combined contributions of feedforward and feedback inputs to bottom-up attention

**DOI:** 10.3389/fpsyg.2015.00155

**Published:** 2015-03-02

**Authors:** Peyman Khorsand, Tirin Moore, Alireza Soltani

**Affiliations:** ^1^Jefferies International Limited, London, UK; ^2^Department of Neurobiology, Stanford University School of Medicine, Stanford, CA, USA; ^3^Howard Hughes Medical Institute, Stanford, CA, USA; ^4^Department of Psychological and Brain Sciences, Dartmouth College, Hanover, NH, USA

**Keywords:** saliency map, saliency computation, top-down attention, computational modeling, feedforward, feedback, lateral interaction, NMDA

## Abstract

In order to deal with a large amount of information carried by visual inputs entering the brain at any given point in time, the brain swiftly uses the same inputs to enhance processing in one part of visual field at the expense of the others. These processes, collectively called bottom-up attentional selection, are assumed to solely rely on feedforward processing of the external inputs, as it is implied by the nomenclature. Nevertheless, evidence from recent experimental and modeling studies points to the role of feedback in bottom-up attention. Here, we review behavioral and neural evidence that feedback inputs are important for the formation of signals that could guide attentional selection based on exogenous inputs. Moreover, we review results from a modeling study elucidating mechanisms underlying the emergence of these signals in successive layers of neural populations and how they depend on feedback from higher visual areas. We use these results to interpret and discuss more recent findings that can further unravel feedforward and feedback neural mechanisms underlying bottom-up attention. We argue that while it is descriptively useful to separate feedforward and feedback processes underlying bottom-up attention, these processes cannot be mechanistically separated into two successive stages as they occur at almost the same time and affect neural activity within the same brain areas using similar neural mechanisms. Therefore, understanding the interaction and integration of feedforward and feedback inputs is crucial for better understanding of bottom-up attention.

## INTRODUCTION

Bottom-up, saliency-driven attentional selection is the mechanism through which the brain uses exogenous signals to allocate its limited computational resources to further process a part of visual space or an object. Early investigations into bottom-up attention showed that this form of attention is fast and involuntary, and purely relies on external inputs that impinge on the retina at a given time (Treisman, 1985; Braun and Julesz, 1998). Therefore, early on, vision scientists hypothesized that bottom-up attention should rely only on parallel, feedforward processes (Treisman and Gelade, 1980; Treisman and Gormican, 1988; Nakayama and Mackeben, 1989). Accordingly, various computational models of attention adopted a similar architecture for bottom-up visual processing (Koch and Ullman, 1985; Wolfe, 1994; Itti and Koch, 2001). More specifically, these models assume that bottom-up attention relies on feedforward processes and computations that terminates in the formation of the saliency (or priority) map, a feature-independent topographical map that represents the visual salience of the entire visual field and can guide covert attention. Nonetheless, all of these models also assume that feedback is involved at some point in visual processing, but this occurs late in processing and only due to top-down signals in tasks which involve top-down attention (e.g., conjunction search, or the search for a target distinguished from other stimuli by more than one feature).

There are a few aspects of bottom-up attentional processes that explain how the hypothesis for the purely feedforward nature of bottom-up attention was originated and why it is still influencing the field, despite more recent contradictory evidence. Specifically, in comparison to top-down attention, bottom-up attention is fast and is relatively unaffected by aspects of the visual stimulus, such as the number of targets on the screen (Treisman and Sato, 1990) or the presence or absence of visual cues (Nakayama and Mackeben, 1989). The relative independence of bottom-up attention from the number of targets is taken as evidence that during bottom-up selection, exogenous signals should be processed in a parallel instead of a serial fashion. Combining this behavioral evidence with the presumption that feedback and recurrent processes are slower than feedforward processes, and that parallel processing excludes feedback, made it appear less likely that bottom-up attention relies on feedback.

However, a number of recent experimental and modeling studies have challenged most of the rather intuitive reasoning mentioned above. On the one hand, there is recent experimental evidence that top-down signals (via inputs to higher cortical areas representing saliency or to lower-level visual areas) can not only alter the previously established behavioral signatures of bottom-up attention (Joseph et al., 1997; Krummenacher et al., 2001; Einhäuser et al., 2008) but also its neural signature (Burrows and Moore, 2009). On the other hand, more recent models of visions have tried to incorporate top-down effects into bottom-up attention in order to design more efficient models of vision that can match human performance in different visual tasks (Oliva et al., 2003; Navalpakkam and Itti, 2005, 2006; see Borji and Itti, 2013 for a review). Importantly, results from a recent biophysically plausible computational model of bottom-up attention, which is mainly concerned with underlying neurophysiological mechanisms, demonstrate that recurrent and feedback inputs do not slow down the saliency computations necessary for bottom-up attention, and instead enhance them (Soltani and Koch, 2010).

Here, we review recent studies that challenge the idea that bottom-up attention solely relies on feedforward processes. Moreover, findings in these studies suggest that mechanistically one cannot separate the feedforward and feedback processes into two successive stages as they occur concurrently and within the same brain areas by using similar neural mechanisms. Therefore, we propose that while thinking in terms of separate feedforward and feedback processes was or maybe is still useful for explaining some behavioral observations, this approach is neither fruitful nor constructive for interpreting the neural data and revealing the neural mechanisms underlying bottom-up attention. Instead, we suggest that understanding the interaction and integration of feedforward and feedback inputs is crucial for understanding bottom-up attention.

## EXPERIMENTAL EVIDENCE FOR THE ROLE OF FEEDBACK

Despite its intuitive appeal, even early studies of attention yielded behavioral evidence against the hypothesis that bottom-up attention relies solely on feedforward processes. This evidence includes, but is not limited to, asymmetries between the search time when targets and distracters are switched (Treisman, 1985), and the impairment of visual search in the presence of a concurrent visual task for the least salient (but not the most salient) target (Braun, 1994). However, these findings were used to argue for parallel versus serial attentional processes and to separate visual processes to “preattentive” (i.e., processes that precede top-down attention and so do not require it) and attentive processes (i.e., processes that require top-down attention; Treisman, 1985; Braun, 1994). That is, instead of assuming a function for feedback in bottom-up attention, they equated feedback processes with the involvement of top-down attention.

The first clear evidence for the role of top-down signals (and therefore feedback) in bottom-up attention comes from a study by Joseph et al. (1997) where they showed that even a visual search for popout targets (which is traditionally considered as a preattentive process) can be impaired in the presence of a demanding central task. Specifically, the authors showed that the performance for detection of an oddball target (defined by a simple feature such as orientation) was greatly impaired when the subjects were simultaneously engaged in reporting a white letter in a stream of black letters. This impairment in performance was alleviated as the lag between the demanding central task and oddball detection was increased, indicating that the impairment was not due to interference between responses in the two tasks. Interestingly, the subjects did not become slower in oddball detection as the number of distracters was increased, a hallmark of parallel processing in visual search tasks. These behavioral results demonstrate that top-down signals are important even for the oddball detection task, which was considered to only rely on preattentive processes, as the shift of such signals to other part of space changes the bottom-up characteristics of performance in the task.

There is other experimental evidence that indicates bottom-up saliency computations are strongly modulated by top-down signals. Some of this evidence is based on inter-trial effects in visual search tasks where the reaction time (RT) for detection of popout targets is influenced by the feature that defined the target on the preceding trial (Maljkovic and Nakayama, 1994; Found and Müller, 1996; Krummenacher et al., 2001, 2010; Mortier et al., 2005). For example, Krummenacher et al. (2001) showed that RT for popout targets was shorter when the feature defining the target on trial “n” was the same as the feature defining the target on trial “n-1.” Because these effects are task-dependent and can survive an inter-trial time interval of a few seconds, it is unlikely that they are caused by activity-dependent changes in the feedforward pathway such as short-term synaptic plasticity which are mostly dominated by depression rather than facilitation (which itself is only prominent on a timescale of a few hundreds milliseconds; Zucker and Regehr, 2002). Overall, these inter-trial effects indicate that not only feedback but also memory can influence bottom-up saliency computations (Krummenacher et al., 2010).

One of the most successful models of bottom-up attention, the saliency model of Itti et al. (1998), assumes the existence of a unique saliency map that represents the visual salience of the entire visual field by integrating saliency across individual features. In order to calculate the most salient locations, the model relies on series of successive computations that separately enhance contrast between neighboring locations for different features of the stimulus such as intensity, orientation, color, motion, etc. This gives rise to the formation of the so-called conspicuity maps for each visual feature which are then further processed and combined to form a single saliency map that has no feature selectivity. This saliency map is proposed to be instantiated in superior colliculus (Kustov and Robinson, 1996), pulvinar (Shipp, 2004), V4 (Mazer and Gallant, 2003), lateral intraparietal cortex (LIP; Gottlieb et al., 1998), or the frontal eye field (FEF; Thompson and Bichot, 2005). Finally, this model assumes that top-down effects could happen via changes at different stages of saliency computations (Itti and Koch, 2001; Navalpakkam and Itti, 2005). Alternatively but not exclusively, top-down effects could directly influence bottom-up attention after the completion of saliency computations (Ahissar and Hochstein, 1997).

There is evidence from viewing (eye movement) behavior that top-down signals can interact with bottom-up saliency signals. In one study, Einhäuser et al. (2008) used a visual search task (using images with manipulated saliency, e.g., by imposing a gradient in contrast across them) to show that task demands can override saliency-driven signals which otherwise bias eye movements. These top-down effects on eye movements could be due to adjustments of weighting of different features involved in saliency computations or direct influence of task demands after bottom-up saliency computations are performed (the so-called weak versus strong top-down effects), or a combination of the two. For example, measuring webpage viewing during different tasks, Betz et al. (2010) argued that the influence of task on viewing behavior could not be merely explained by reweighing of features. Whereas effects of task demands on viewing behavior are present in both of these studies, the exact locus and relative contribution of top-down signals to bottom-up processes could depend on the task (e.g., visual search versus information gathering from texts). Moreover, it is more biophysically plausible (in terms of existing feedback connections and neural circuitry) that top-down signals and task demands exerts their effects on bottom-up attention via modulating the saliency computations as they progress, rather than overriding the final computations.

Overall, these behavioral results demonstrate that bottom-up saliency computations (e.g., detecting an oddball) are strongly modulated by feedback signals and processes that include working memory. Moreover, they provide an alternative way to interpret the aforementioned asymmetries in the detection of a salient object, or, the dichotomy between preattentive and attentive processes. That is, the detection of any target (salient on non-salient) requires some amount of feedback from higher visual areas; however, the necessary amount of feedback depends on the configuration of targets and distracters (see below).

Despite earlier behavioral evidence for the role of top-down signals in bottom-up attention, the corresponding neural evidence has been demonstrated only recently (Burrows and Moore, 2009). More specifically, Burrows and Moore (2009) examined the representation of salience in area V4, as previous attempts at finding these signals in lower visual areas were equivocal (Hegdé and Felleman, 2003), and moreover, examined the effects of top-down signals on this representation. In order to distinguish pure salience signals from signals that merely reflect a contrast between the center and surround (such as orientation contrast reported by Knierim and van Essen, 1992), the authors measured the response of V4 neurons to different types of stimuli (singleton, color and orientation popout, combined popout, and conjunction), for which the target has different levels of saliency. Interestingly, they found that V4 neurons carry pure saliency signals reflected in their differential firing responses to popout and conjunction stimuli. Next, they measured the response to the same stimuli while a monkey prepared a saccade to a location far from a neuron’s receptive field. Interestingly, they found that saccade preparation eliminated the saliency signals observed in V4. Later, our computational modeling showed that these observations can be explained by alterations of feedback from neurons in a putative saliency map due to saccade preparation (Soltani and Koch, 2010). Overall, these results demonstrate that the most basic computations underlying bottom-up attention, which enable the brain to discriminate between salient and non-salient objects, are strongly modulated by top-down signals.

## MODELING EVIDENCE FOR THE ROLE OF FEEDBACK

Most computational models of bottom-up attention rely on feedforward processes as the main source of computations during visual search tasks (Koch and Ullman, 1985; Treisman and Gormican, 1988; Wolfe, 1994; Itti and Koch, 2001). Whereas some of these models were constructed keeping neural substrates in mind, they lack enough detail to be able to elucidate biophysical mechanisms or constraints underlying bottom-up attention. As described below, some of these biophysical constraints are the main reasons why feedforward processes are not sufficient to adequately account for the behavioral and neural signatures of bottom-up attention.

In a recent study, Soltani and Koch (2010) constructed a detailed, biophysically plausible computational model to examine neural mechanisms and constraints underlying the formation of saliency signals. The model network consisted of populations of spiking model neurons representing primary visual areas (V1, V2, and V4) and a higher visual area representing the saliency (or priority) map, a topographical map that represents the visual salience of the entire visual field. Similar to the saliency model of Itti et al. (1998), Soltani and Koch (2010) assumed that the neural population in the saliency map integrates the output of neural populations in V4 with different features selectivity. Therefore, the saliency signals in visual areas V1–V4 were feature-dependent whereas this signal was feature-independent in the saliency map. The input to the model was generated by filtering stimuli used in Burrows and Moore’s (2009) study based on response properties of neurons in LGN and V1. Using this model, the authors studied both the formation of saliency signals in successive populations of neurons (which mimic visual areas V1–V4) and how these signals are modulated by the feedback from a putative saliency map (assumed to be instantiated in the LIP or FEF).

The results from this computational study challenge the idea that bottom-up, exogenous attention solely relies on feedforward processing at various levels. Firstly, this study provides evidence that saliency processing relies heavily on recurrent connections (so it is not solely feedforward) with slow synaptic dynamics operating via NMDA receptors. However, the involvement of NDMA-mediated currents does not slow down the emergence of saliency signals. More specifically, the onsets of saliency signals in successive layers of the network were delayed by only a few milliseconds (and were advanced for some stimuli), while the strength of signals greatly increased. Secondly, as shown experimentally and computationally, recurrent reverberation through NMDA is crucial for working memory (Wang, 1999; Tsukada et al., 2005; Wang et al., 2013) and decision making (Wang, 2002). Therefore, an equally important role for reverberation through NMDA in saliency computations makes these computations more similar to cognitive processes that are not considered feedforward, such as working memory and decision making. Thirdly, this study demonstrates that whereas saliency signals do increase across successive layers of neurons, they could be significantly improved by feedback from higher visual areas that represent the saliency map.

But how is it that recurrent and feedback inputs do not slow down saliency computations in the model? The formation of saliency signals relies heavily on slow recurrent inputs (dominated by NMDA receptors), but at the same time these signals propagate through successive layers of the network via fast AMPA currents. Computation at successive layers with slow synapses reduces noise and enhances signals such that higher visual areas carry the saliency signals earlier than the lower visual areas. Consequently, feedback from the higher visual areas via fast AMPA synapses can enhance the saliency signals in the lower visual areas. Importantly, all these results depend on the presence of cortical noise. In the absence of noise, saliency computations could be accomplished merely by AMPA currents and do not require successive layers of neural populations (as in the saliency model of Itti et al., 1998).

Another important aspect of modeling results is that due to noise and basic mechanisms for saliency computations (i.e., center-surround computations via lateral interaction) the optimal architecture for these computations is for them to process visual inputs in separate populations of neurons selective for individual features. This feature could explain the inter-trial effects similarly to the parallel coactivation model of Krummenacher et al. (2001). In that model, the feature of the target on the preceding trial could deploy top-down attention to enhance processing in population selective to that feature, therefore, decreasing RT for the “same” versus “different” trials. In our model, the saliency signals in a population selective to the repeated feature could be enhanced due to feedback signals (caused by working memory of previously selected target), while the same feedback increases noise in the non-repeated population and results in a slower RT. Despite this advantage for separate processing of various features, future studies are required to explore the role of neural populations with mixed selectivity in saliency computations.

**FIGURE 1:**
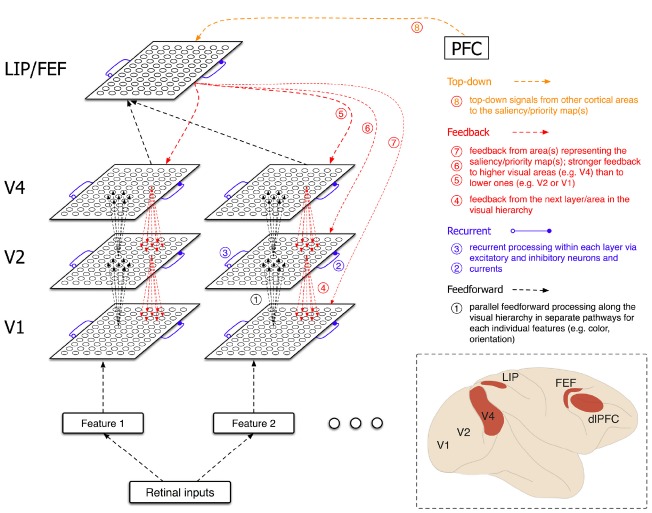
**Schematic of the network architecture and different types of neural processes (feedforward, recurrent, feedback, and top-down) involved in bottom-up attention (saliency computations).** Saliency computations start with the process of external inputs that fall on the retina. *Feedforward* processing of the inputs, in separate pathways selective to different visual features (color, orientation, etc.), in successive layers of neural populations (from V1 to V4) enhances the signals that could guide attentional selection. However, this enhancement requires interactions between neighboring neurons via *recurrent* excitatory and inhibitory inputs. Because the saliency signals become stronger in successive layers, *feedback* from the next layer/area in the visual hierarchy could further enhance the signals. Ultimately, outputs of different pathways are combined to instantiate the saliency/priority map(s) (possibly in area LIP and/or FEF) that represents the visual salience of the entire visual field and can determine the next attended location. *Feedback* from the saliency/priority map(s) to lower visual areas could further enhance the saliency signals. Moreover, *top-down* signals from other cortical areas such as dlPFC could exert top-down effects and task demands on saliency computations. The inset shows a cartoon of macaque’s brain with relevant areas highlighted.

In the aforementioned model, only feedback from neurons in the saliency map to those in early visual areas was considered. However, we propose a more general form of feedback that also includes feedback between visual areas (from the next layer/population) as well as top-down signals from other cortical areas to the saliency map(s) (Figure 1). Moreover, because the projections that mediate feedback are active whenever the presynaptic neurons are active, independently of the task demands, the feedback is always present (unless top-down signals suppress these activities at their origin) and exerts their effects on visual processes. Considering the short delays in transmission of visual signals across brain areas, separating bottom-up attentional processes into feedforward and feedback components could be mechanistically impossible.

There are high-level models of bottom-up attention that address the influence of top-down signals on bottom-up attention in general (see Borji and Itti, 2013 for a review) or for improving object recognition (Oliva et al., 2003; Navalpakkam and Itti, 2005, 2006). In some of these models, top-down effects are simulated via multiplicative gain modulations of bottom-up computations (Navalpakkam and Itti, 2006) or as an abstract term (contextual priors) in computing the posterior probability of an object being present (Oliva et al., 2003). However, in most computational models of visual attention that strive to predict the pattern of eye movements in real time, the distinction between top-down and bottom-up processes are not clear (Borji and Itti, 2013). Importantly, the main result of those modeling works is that top-down signals are crucial to achieve performance that matches human visual performance and can accurately predict eye movements. However, because of the high-level nature of these models, computations performed by these models are not constrained and so are not biophysically plausible. Therefore, these models do not elucidate biophysical constraints underlying bottom-up attention that could reveal the role of feedback on bottom-up attention. Perhaps, the lack of distinction between bottom-up and top-down processes in more advanced models of visual attention is an indication that one cannot separate these processes based on behavior alone.

## MORE EVIDENCE FOR THE ROLE OF FEEDBACK: SPEED OF FEEDFORWARD, RECURRENT, AND FEEDBACK PROCESSES

As described above, one of the reasons for assuming that bottom-up attention relies on feedforward processes is because feedback and recurrent inputs are not fast enough, e.g., by considering the time it takes for the visual signals to travel from lower to higher visual areas and back. Nevertheless, as shown by the recent modeling work, even recurrent inputs through slow NMDA synapses do not impede the emergence of saliency signals in successive layers of neural populations, and feedback can enhance those signals (Soltani and Koch, 2010). Interestingly, compatible with the model’s assumptions and predictions, there is growing neurophysiological evidence that feedback and recurrent inputs actually do contribute to bottom-up attention (see below).

As shown by computational models with different levels of detail, saliency computations heavily rely on center-surround computations (Itti and Koch, 2001). One prevalent form of center-surround computations recorded neurophysiologically is the surround suppression (i.e., suppression of response by stimuli outside the classical RF (CRF); Cavanaugh et al., 2002). Surround suppression is observed even in the primary visual cortex as well as retinal ganglion (Kruger et al., 1975) and LGN cells (Levick et al., 1972), and has been assumed to be instantiated by horizontal connections from neighboring neurons with similar selectivity. However, by analyzing the timing of surround suppression and how it depends on the distance of the stimuli outside the CRF, Bair et al. (2003) found not only that the latency of suppression depends on its strength but also that this suppression could arrive faster than the excitatory CRF response and does not depend on the distance of the surround stimuli. To explain these results, Bair et al. (2003) suggested that in addition to recurrent inputs, surround suppression in V1 might be strongly influenced by feedback from higher visual areas (e.g., V2) with a larger RF. In another experiment, Hupe et al. (1998) found that feedback from higher visual areas (area V5) is crucial for surround suppression within early visual areas (V1, V2, and V3). More specifically, the authors showed that inactivation of V5 greatly reduces surround suppression in V3 neurons. These findings corroborate the idea that even the simplest form of saliency computations depends on feedback which could enhance the speed of computations within the same layer simply because feedback connections are faster than horizontal connections by an order of magnitude (Bringuier et al., 1999; Girard et al., 2001).

While feedforward connections are faster than horizontal connections, it is known that feedforward and feedback connections are equally fast (about 3.5 m/s) and have latencies as short as 1.5 ms (Girard et al., 2001). Therefore, feedback processing can be as fast as feedforward processing, but with the advantage that higher visual areas carry larger saliency signals as shown experimentally and computationally (Hegdé and Felleman, 2003; Burrows and Moore, 2009; Soltani and Koch, 2010; Bogler et al., 2011; Melloni et al., 2012; see below). Interestingly, the difference in the response latency in different visual areas can be very small, while the represented signals can be very different at different time points. For example, Bisley et al. (2004) showed that the visual response in LIP could emerge as quickly as 40 ms, which matches the latency of the visual response in the primary visual cortex. This could happen by bypassing successive processing of visual information (Schmolesky et al., 1998), and indicates that signals from salient targets may emerge in higher visual areas very quickly.

## CONTRIBUTION OF DIFFERENT BRAIN AREAS TO SALIENCY COMPUTATIONS

Whereas early studies that investigated the neural representation of bottom-up attention found saliency signals in early visual areas such as V1 (Knierim and van Essen, 1992), later studies showed that distinct saliency signals are only present in higher visual areas (Hegdé and Felleman, 2003; Burrows and Moore, 2009; Betz et al., 2013). As mentioned earlier, electrophysiological studies were able to distinguish pure salience signals from signals that merely reflect a contrast between the center and surround by measuring the response of neurons to different types of stimuli for which the target has different levels of saliency (Hegdé and Felleman, 2003; Burrows and Moore, 2009).

Using a similar approach, recent fMRI studies indicate that saliency signals emerge gradually over successive brain areas. For example, an attempt to identify how saliency signals progress through the brain demonstrated that while activity in early visual areas is correlated with the graded saliency in natural images, activity in higher visual areas (such as anterior intraparietal sulcus and the FEF) is correlated with the signal associated with the most salient location in the visual field (Bogler et al., 2011). The latter observation supports the idea that a winner-take-all mechanism results in selection of the most salient location only in higher visual areas. Another recent study showed the gradual emergence of bottom-up signals in early visual areas (Melloni et al., 2012). Specifically, considering a “TSO-DSC” stimulus (a stimulus that contains a target that was singleton in orientation but also contains a highly salient distractor in a task-irrelevant dimension) as a conjunction stimulus, the patterns of activation across successive visual areas are similar to the results from the computational model of Soltani and Koch (2010). That is, only in V4, the response to both types of popout is larger than the response to the conjunction stimulus. Finally, compatible with what Burrows and Moore (2009) reported, an fMRI study found that in the presence of a demanding central task, saliency signals (in the form of orientation popout) are only present in higher visual areas (V3 and V4) and not in V1 (Bogler et al., 2013).

Despite strong neural evidence for the instantiation of saliency map in higher cortical area, it has been argued that the saliency map could be represented by V1 neurons (Li, 2002). The support for this proposal has been mainly based on behavioral data, but a recent fMRI study has provided some neural evidence for saliency signals (in the absence of awareness) in V1–V4 and not higher cortical areas (Zhang et al., 2012). However, using stimuli for which saliency and luminance contrast were uncorrelated, a more recent study showed that most BOLD activity in early visual areas (V1–V3) is dominated by contrast-dependent processes and does not comprise contrast invariance which is necessary for saliency representation (Betz et al., 2013).

Moreover, instantiation of saliency map in early visual areas is not very feasible and imposes serious constraints for saliency computations and the observed effects of top-down signals. Firstly, area V1 is not well-equipped for performing saliency computations. For example, V1 neurons lack certain feature selectivity and therefore, saliency computations in V1 imply that those features cannot contribute to saliency and bottom-up attention. Secondly, saliency computations (center-surround computations, pooling of signals over different features) eliminate some of the information presents in V1 and therefore, limiting information processing that higher visual area can perform on the output of V1. Thirdly, feedback projections to V1 are not very strong and this significantly limits the effects of top-down signals on saliency computations. Finally, our computational results show that saliency computations require successive processing of visual information over multiple layers and cannot be replaced by a stronger interaction within one layer of neural population (Soltani and Koch, 2010). For these reasons, we think that instantiation of a real saliency map in V1 is not plausible.

As mentioned earlier, the observed asymmetries in popout detection reveal the importance of feedback in bottom-up attention. For example, the finding of Schiller and Lee that lesions of V4 differentially affect detection of the most and least salient targets (Schiller and Lee, 1991; which was used by Braun, 1994 as evidence for different attentional strategies) could indicate that detection of any target requires feedback. More specifically, in the case of detecting the least salient target, feedback from higher visual areas is required to suppress the activity in most parts of the visual space, a process that could be easily interrupted by V4 lesions. On the other hand, detection of the most salient target requires only feedback that enhances activity in the target location, a process that could be only mildly disrupted by V4 lesions.

Whereas we mainly discussed the modulation of bottom-up attention by top-down signals via their effects on early visual areas, there is experimental evidence that even activity in the putative saliency map is modulated by top-down signals (Thompson et al., 2005; Ipata et al., 2006; Suzuki and Gottlieb, 2013). In one study Thompson et al. (2005) showed that during a popout search task, where target and distractor colors switched unpredictably, monkeys made more erroneous saccades to distracters on the first trial after the switch. Importantly, presaccadic neural activity in the FEF was informative about the selected stimulus independently of whether the stimulus was a popout target or one of many distracters. Moreover, the signal conveyed by FEF neurons was correlated with the probability that a given target would be selected, indicative of this area to instantiate the saliency map. In another study Ipata et al. (2006) trained a monkey to ignore the presentation of a popout distracter during a visual search task, while they recorded from LIP neurons. They found that on trials where the monkey ignored the distracter, the LIP response to the salient distracter was smaller than the response to a non-salient distracter. Recently, Suzuki and Gottlieb (2013) compared the ability of LIP and dorsolateral prefrontal cortex (dlPFC) in suppressing distracters using a memory saccade task where a salient distracter was flashed at variable delays and locations during the memory delay. Interestingly, they found that not only dlPFC neurons showed stronger distractor suppression than LIP neurons, but also reversible inactivation of dlPFC gave rise to larger increases in distractibility than inactivation of LIP. Overall, these results show that even the activity of neurons in the putative saliency map is modulated by top-down signals and moreover, these signals strongly contribute to performance in attention tasks.

Considering strong projections from areas representing the putative saliency map (LIP/FEF) to lower cortical areas (Blatt et al., 1990; Schall et al., 1995) and the fact that this feedback is present as long as the former areas are acitve (in both correct and incorrect trials), one can predict specific effects of activity in the saliency map on neural processes in lower visual areas. Interestingly, the modeling results described above indicate that the main reason for a concurrent task (or even saccade planning as in Burrows and Moore) interfering with bottom-up computations is the influence of the concurrent task on the activity in the saliency map (LIP or FEF). This happens because the bump of activity from planning a saccade suppresses neural activity in most parts of the saliency map except the saccade location, interrupting and altering feedback from neurons in those parts of the map. The behavioral results for detecting two popout targets at various distances show that the RT redundancy gain (shortening of RT when popout is defined by two features compared to when it is defined by one feature) decreases as the distance between the two targets increases (Krummenacher et al., 2002). This may be explained by the fact that two bumps of activity in the saliency map interact weakly if they are too far from each other (or alternatively due to interactions in early visual areas, which is less likely due to weaker interactions between neurons selective to different features in these areas). On the other hand, at short distances these bumps compete (with higher probability of winning for the faster detected (more salient) target) resulting in an increase in feedback based on the most salient location and therefore higher RT gains. Future experiments are needed to study the effects of inter-trial variability of neural responses in higher cortical areas on bottom-up attentional processes in lower visual areas.

## SIMILARITIES BETWEEN BOTTOM-UP AND TOP-DOWN ATTENTION

Considering that top-down attention likely involves feedback inputs, examining similarities between bottom-up and top-down attention can further shed light on the role of feedback in bottom-up attention. These include similarities in: the timing of bottom-up and top-down attentional signals in different brain areas; neural substrates of bottom-up and top-down attention; and involved neurotransmitters.

Importantly, a few studies have examined the timing of bottom-up and top-down attentional signals in different brain areas. In one study, Buschman and Miller (2007) found earlier bottom-up signals in LIP than in lateral prefrontal cortex and the FEF whereas FEF neurons detected conjunction targets before LIP neurons. Other studies, however, point to a more complicated formation of attentional signals in prefrontal and parietal cortices. For example, a recent study by Katsuki and Constantinidis (2012) showed that neurons in dlPFC and posterior parietal cortex signal bottom-up attention around the same time. Interestingly, there is evidence that top-down attentional enhancements of activity within visual cortices are larger and earlier in higher areas (V4) compared to lower areas (V1), indicative of a “backward” propagation of modulatory signals (Mehta et al., 2000a,b; Buffalo et al., 2010). Moreover, the laminar source of attentional modulations in primary visual cortices supports the idea that feedback from the next visual area in the hierarchy is the origin of these modulations (Mehta et al., 2000a,b). This is compatible with the finding that during top-down attention, the FEF neurons exhibit attentional modulation about 50 ms before V4 neurons (Gregoriou et al., 2009). These observed trends of neural modulations resemble successive processing of bottom-up attentional signals, and earlier emergence of saliency signals in higher visual areas.

Interestingly, even the timing of top-down attentional signals could be similar between the lower and higher visual areas. A recent study found that signals related to object-based attention can be detected in primary visual areas and the FEF at the same time (by simultaneous recording from V1 and the FEF), and that the interaction between these areas determines the dynamics of target selection (Pooresmaeili et al., 2014). These observations challenge the feedforward assumption behind the formation of bottom-up attentional signals and point to the role of reciprocal interactions within lower and higher visual/cortical areas. An interesting aspect of the observed neural response in the FEF (which was not present in V1) was an increase in the differential response to target and distracter over time, indicative of a winner-take-all process in the FEF. Comparing recordings from V1 and the FEF, which seem to reside on the opposite sides of visual hierarchy for visual attention, shows that while the visual response in V1 occurs earlier than in the FEF, the selection signal occurs at the same time in both of these areas (Khayat et al., 2009). However, the modulation index of neuronal response in area V1 was much smaller than the one in the FEF indicating more enhanced signals in the latter area. Interestingly, Khayat et al. (2009) also found that on error trials FEF activity precedes V1 activity and therefore imposes its erroneous decision. These results show the important role of ever-present feedback from higher cortical areas in object-based attention.

Another piece of evidence supporting similarities between neural substrates underlying bottom-up and top-down attention comes from two separate experiments measuring the effects of FEF microstimulation on information processing in other visual areas. Considering the FEF as a higher visual area that controls top-down attention, one would assume that its microstimulation would enhance visual signals in lower visual areas that show attentional modulations, independently of bottom-up driven signals in the latter areas. However, using different methods for measuring signals (single cell recordings and fMRI), two separate experiments found that induced enhancements of visual signals depended on the already present bottom-up signals. In one study, Moore and Armstrong (2003) found an increase in spiking activity in V4 only when a target was present in the V4 RF, and this enhancement was larger in the presence of a competing distracter. In another study, in which changes in fMRI BOLD responses throughout visual cortex were measured, Ekstrom and Roelfsema (2008) found that the effect of FEF microstimulation on posterior visual areas (such as V4) depends on the stimulus contrast and the presence of distracters. These results demonstrate that even artificially simulated top-down effects are not independent of bottom-up saliency signals, which renders the distinction between feedforward and feedback processes even more unnecessary.

The modeling results also predicted that saliency computations should rely on excitatory and inhibitory recurrent inputs within each layer of neural populations and the excitatory recurrent input should be dominated by NMDA receptors (and not AMPA receptors), in order to integrate saliency signals in the presence of cortical noise (Soltani and Koch, 2010). There is recent experimental evidence supporting this prediction. In one study, Self et al. (2012) used different drugs to measure the contribution of AMPA and NMDA receptors to figure-ground modulations (the increased activity of neurons representing the figure compared with the background) in V1. They found that AMPA currents mainly contribute to feedforward processing and not to the figure-ground modulations, whereas NMDA blockade reduces figure-ground modulations. Another recent study showed that NMDA, and not AMPA, receptors contribute to the reduction of variance and noise correlation due to attention (Herrero et al., 2013). Both these results corroborate the modeling results that NMDA receptors are crucial for saliency computations and bottom-up attention.

Interestingly, NMDA receptors are modulated by dopamine (Cepeda et al., 1998; Seamans et al., 2001), the main neurotransmitter for signaling reward that also influences working memory (Williams and Goldman-Rakic, 1995) and can alter visual processes on a long timescale (Bao et al., 2001). However, in the absence of strong dopaminergic projections to primary visual cortex (Lewis and Melchitzky, 2001), most dopamine-dependent modulations of visual processing may occur via dopamine effects on prefrontal activity and resulting modulated feedback. For example, recent studies found that dopamine effects on the FEF activity can enhance the visual response in V4 neurons (Noudoost and Moore, 2011) and contribute to adaptive target selection (Soltani et al., 2013) via specific types of receptors. Considering the effects of dopamine on working memory and the fact that top-down attention requires some forms of working memory, one might regard dopamine as the primary neuromodulator for top-down attention. However, one needs to exercise caution because of the aforementioned evidence for the role of feedback in bottom-up attention suggesting that dopamine could have a significant role in bottom-up attention.

In summary, the role of NMDA in saliency computations highlights shared neural substrates for bottom-up attention and cognitive processes that are not considered feedforward (such as working memory and decision making). Moreover, the strong effects of neuromodulators on NMDA receptors indicates how various neuromodulators could affect bottom-up attention via their effects on higher visual areas that provide feedback to early visual areas, or by directly altering saliency computations.

The aforementioned similarities between bottom-up and top-down attentional processes removes a clear distinction between neural substrates of bottom-up and top-down attention based on the location of a given area in the visual hierarchy, and point to a stronger role of feedback in bottom-up attention. Moreover, similarities between bottom-up and top-down attention, which are originally assumed to rely on feedforward and feedback inputs, respectively, indicate that both these inputs are important for both types of attention. These observations signify that the main dichotomy of visual attention should be disregarded in the search of more unified models of attention.

Recently, Awh et al. (2012) have elegantly challenged the bottom-up and top-down dichotomy and instead proposed a framework that relies on a priority map that integrates multiple selection mechanisms and biases including: current goals, selection history, and physical salience. Specifically, they summarized experimental evidence supporting the idea that both recent history of attentional deployment as well as the reward history can bias visual selection independently of the current goals (top-down signals) or stimulus salience (bottom-up signals). Interestingly, the main argument of Awh et al. (2012) for the failure of attentional dichotomy is unexplained selection biases due to lingering effects of past experience, either selection history and reward history. However, the only feasible mechanism for the effects of past experience on attentional selection could be synaptic plasticity, if one wants to truly separate mechanisms underlying these effects from those serving the influence of top-down signals (due to some sustained activity in some higher cortical areas). Because of different timescales of selection history and reward history, their effects should rely on short-term and long-term synaptic plasticity, respectively. This has important implications for the effects of neuromodulators on bottom-up attention.

## DISCUSSION AND FUTURE DIRECTIONS

We have reviewed the experimental and modeling evidence for the role of feedback in bottom-up attention. From a behavioral point of view, there is evidence that top-down signals are necessary for observing characteristics of bottom-up attentional processes such as the very fast detection of oddball targets. From a neuronal point of view, signals reflecting the saliency of an object can be diminished when top-down signals are interrupted, for example during saccade preparation. From a computational point of view, bottom-up, saliency computations can be enhanced by feedback from higher visual areas that represent the saliency map. Considering this evidence, it may be logical to replace bottom-up attention with salience-dependent attention, as the former term implies a specific direction for information processing which is not compatible with most experimental or computational results.

As suggested by Awh et al. (2012), some of the experimental findings reviewed here can be considered as the lingering effects of past experience on attentional selection (due to recent history of attentional deployment). This includes inter-trial effects on performance and RT (e.g., work of Krummenacher and colleagues). On the other hand, effects of reward history on attentional selection are not discussed here but are of great importance for understanding attentional processes. Both their and our proposals challenge the bottom-up versus top-down dichotomy, but point to different mechanisms that could account for unexplained observations. More specifically, Awh et al. (2012) point to the role of short-term and long-term synaptic plasticity in the feedforward pathways to explain some of the observed selection biases. In contrast, we assign an important role for feedback between successive stages of saliency computations and from the saliency map(s) to lower visual areas, as well as interaction and integration of top-down and bottom-up signals within the saliency map(s). While these mechanisms are not exclusive, future work is needed to clarify the specific role and relative contribution of them in attentional selection.

Overall, the reviewed findings indicate that in order to reveal the neural substrates of attentional processes, the focus should be shifted toward understanding biophysical mechanisms through which the necessary computations could be performed, and whether a specific brain area has the proper neural type and connectivity to perform those computations. Therefore, even though the results described above reduce the role of unidirectional, hierarchal computations (i.e., from lower to higher visual areas) and minimize the distinction between feedforward and feedback inputs in bottom-up attention, one should not ignore the anatomical and biophysical constraints underlying these computations. For example, whereas successive processing across visual hierarchy can be bypassed, area V4 still sends more projections to higher visual areas (such as the FEF) than area V1 (Schall et al., 1995). On the other hand, the projections from the FEF to area V4 mostly target pyramidal neurons in primary visual areas (Anderson et al., 2011). The observed lack of projections to inhibitory neurons limits mechanisms through which feedback projections could exert modulatory effects, instead of just driving the recipient areas. Understanding the implications of these and other constraints on feedforward and feedback processing could provide valuable insight into understanding bottom-up attention in particular and vision in general.

There are still many unanswered questions about the role of feedback in bottom-up, exogenous attention. Firstly, while the benefit of feedback from a higher visual area representing the saliency map has been established, there is need for further research that investigates the effects of feedback between each successive layers/areas using detailed computational models. Secondly, the saliency signals are observed in many brain areas (FEF, LIP, superior colliculus, dlPFC), all of which provide feedback to early visual areas. This indicates that there should be interaction between these signals in order to deploy attention to a unique location; understanding this interaction is crucial for understanding bottom-up attention. Interestingly, some of these areas contribute to top-down attention, which requires working memory, and it is important to see how saliency and working memory signals interact and integrate within the saliency/priority maps. Thirdly, all primary visual areas receive feedback from higher visual areas representing the saliency map. Future computational work is needed to elucidate the relative contribution of feedback to a specific brain area (e.g., V1 in comparison to V4). Overall, considering the complexity of behavioral and neural data, more detailed computational models are needed to study interaction and integration of feedforward and feedback inputs in order to provide a more coherent account of bottom-up attention and its underlying neural mechanisms.

As experimental methods for manipulations and simultaneous measurements of neural activity improve, there is a greater need for more extensive and detailed computational models to interpret the outcome data and provide predictions for future experiments. Future experiments with simultaneous recording of neural activity should allow us to study the relationship between feature selectivity (tuning) and saliency signals for individual neurons. Computational models are needed to explain such relationships and how different neural types contribute to bottom-up attention. Similarly, drug manipulations of various brain areas provide another opportunity for computational modeling to contribute, considering the large number of involved receptors and contradictory possible outcomes (e.g., Disney et al., 2007; Herrero et al., 2008).

### Conflict of Interest Statement

The authors declare that the research was conducted in the absence of any commercial or financial relationships that could be construed as a potential conflict of interest.
